# A Therapeutic Conversational Agent (Solace) for Management of Chronic Pain: Acceptability and Usability Study

**DOI:** 10.2196/87689

**Published:** 2026-03-20

**Authors:** P Maxwell Slepian, Stephanie Buryk-Iggers, Anna M Lomanowska, Binh Nguyen, Tahir Janmohamed, Hance Clarke, Joel Katz, Nils G Niederstrasser

**Affiliations:** 1 Department of Anesthesia and Pain Management University Health Network Toronto, ON Canada; 2 Department of Anesthesiology and Pain Medicine Temerty Faculty of Medicine University of Toronto Toronto, ON Canada; 3 University of Toronto Centre for the Study of Pain University of Toronto Toronto, ON Canada; 4 ManagingLife, Inc Toronto, ON Canada; 5 Department of Psychology Faculty of Health York University Toronto, ON Canada; 6 School of Psychology, Sport, and Health Sciences University of Portsmouth Portsmouth, England United Kingdom

**Keywords:** artificial intelligence, chatbot, chronic pain, digital health, pain psychology

## Abstract

**Background:**

Chronic pain is a critical cause of personal suffering and societal concern. However, treatment options remain inadequate, and access to efficacious treatment is limited by geography, economics, and scale. Digital health interventions for chronic pain are easily scaled solutions to this problem, and autonomous conversational agents represent a new frontier in this treatment domain. Despite their potential impact, conversational agents powered by generative artificial intelligence (AI) have yet to be developed or examined for the treatment of chronic pain.

**Objective:**

We aimed to develop and test Solace, a first-of-its-kind, expert-trained generative AI conversational agent designed to deliver support grounded in principles of evidence-based pain psychology.

**Methods:**

We conducted an acceptability and usability study of Solace in a group of individuals with chronic pain. Participants (n=175) were recruited from Prolific, an online crowdsourcing platform, and interacted with Solace for at least 25 minutes. Self-report measures of system usability, treatment acceptability, and therapeutic alliance were completed after the interaction, and clinically relevant pain-related measures were completed before and after the interaction.

**Results:**

Participants rated the usability of Solace to be excellent (System Usability Scale mean score 85.04, SD 13.6) and found it to be an acceptable intervention for chronic pain. The therapeutic alliance between participants and Solace was rated highly. Participants also demonstrated improvements in anxiety, pain interference, kinesiophobia, and pain resilience. Safety guardrails designed to identify and manage instances of suicidal ideation, injury, or requests for medication recommendations performed appropriately during the study. Solace is a usable and acceptable treatment for chronic pain that facilitates forming a strong therapeutic alliance with users. A single 30-minute conversation with Solace was also associated with improvements in several clinically relevant domains.

**Conclusions:**

Solace is a usable and acceptable expert-trained generative AI conversational agent for pain management. Randomized clinical trials are needed to evaluate the efficacy of Solace as a strategy for the treatment of chronic pain.

**Trial Registration:**

OSF Registries osf.io/9c7tv; https://osf.io/9c7tv

## Introduction

Chronic pain affects approximately 20% of adults worldwide, and is an enormous source of personal suffering as well as a major public health challenge [1,2]. Psychological interventions are efficacious for chronic pain. Across numerous large-scale randomized controlled trials, cognitive behavioral therapy, in particular, has been robustly associated with improvements in pain intensity and pain-related disability [3]. There is also accumulating evidence that other psychological treatment approaches, including mindfulness-based interventions and acceptance and commitment therapy (ACT), are similarly effective [4-7].

Despite the wealth of evidence for pain psychology interventions, widespread implementation of these interventions has been limited. This may be due, in part, to systemic barriers to adoption, including insufficient finances, time constraints, poor accessibility, and social stigma [8,9]. However, one of the key limitations is simply the lack of competent providers. For instance, difficulty accessing competent providers is the most common barrier reported by individuals with chronic pain [8]. This is further complicated by the fact that most licensed health care providers practicing therapy (eg, psychiatrists, psychologists, counselors, and social workers) report little to no education or training in pain treatment [8].

Digital health interventions (DHIs) have emerged as one of the primary means to overcome these barriers and bring psychological interventions for pain to scale [10,11]. DHIs exist in many forms, ranging from distance-based replications of services via telephone or videoconferencing to web-based treatment programs and mobile applications. Across these services, content can be delivered in either a fully self-guided format or supported by a provider or “coach,” either synchronously or asynchronously [12]. These interventions have broadly demonstrated efficacy, but uptake remains limited, with studies reporting high dropout rates and low adherence [10,13].

More recently, researchers have begun to examine the provision of therapy by conversational agents, or chatbots, that enable patients to have interactive conversations in real time [13]. A recent scoping review identified beneficial effects of chatbots on pain intensity, but results were equivocal for pain interference and mental health outcomes [13]. These chatbots have generally been designed according to rule-based response algorithms rather than large language model (LLM)–based conversational support [13-16]. Such approaches have demonstrated that conversational agents can build a strong therapeutic alliance with users [15].

The use of LLM-based generative artificial intelligence (AI) conversational agents offers an alternative to algorithm-based chatbots. To date, not one study has examined fully generative AI conversational agents for the treatment of chronic pain. However, such generative AI chatbots have been studied for general mental health treatment. A clinical trial of a generative AI chatbot, Therabot*,* identified clinically significant improvement in symptoms of depression, anxiety, and feeding/eating disorder risk after 4 and 8 weeks, compared to waitlist controls [17]. Generative AI chatbots are also being applied to coping with medical concerns. In a recent study, health care providers identified a generative AI chatbot designed to provide coping support for adolescents with cancer as a feasible option for support provision, although the impact on clinical outcomes remains to be examined [18].

We sought to develop and test Solace, an expert-trained, generative AI chatbot built by ManagingLife, Inc. Solace was created by pairing a purpose-built knowledge base with a multiagent architecture leveraging a variety of commercially available foundational models. This study describes a prospective feasibility evaluation of the acceptability and usability of Solace in a group of participants reporting chronic pain (n=175). Participants engaged in an open-ended 30-minute interaction with Solace and completed measures of treatment acceptability, system usability, and therapeutic alliance after the conversation. Pain-related self-report measures were also completed before and after the participant’s interaction with Solace. We hypothesized that individuals would find Solace to be a usable and acceptable treatment for chronic pain.

## Methods

### Study Design

This study was a prospective feasibility evaluation [19,20] of the AI-driven conversational agent, Solace*.* The design was guided by the Developmental and Exploratory Clinical Investigation of Decision-Support Systems Driven by Artificial Intelligence (DECIDE-AI), providing an early-stage clinical evaluation checklist of AI-specific reporting items (Multimedia Appendix 1) [21].

### AI Chatbot

#### Overview

Solace is a stand-alone, web-based digital health application developed by ManagingLife [22]. It interacts with users to gather information related to the user’s experience of pain and delivers appropriate, structured, interactive support grounded in evidence-based pain psychology techniques (eg, psychoeducation and ACT) to bolster emotional coping and self-management in individuals living with chronic pain. The development process focused on ensuring appropriate psychological support for adults with chronic pain through systematic programming and testing phases.

#### Technical Infrastructure

This study used the Solace v1.0 beta system version. The technical backbone of the chatbot system was built on top of a cloud-based digital health platform, Manage My Pain, developed by ManagingLife [22]. The main components of the Manage My Pain platform included user authentication, secure cloud hosting, and compliance with international data privacy regulations, including end-to-end encryption and role-based access controls. Solace was designed to extend this platform by leveraging Amazon Web Services Bedrock to build a multiagent architecture using a library of LLMs. Each agent is responsible for a specialized task, and, together, these agents are coordinated by a centralized orchestration layer that manages conversational flow, memory retention, response timing, and system-level safety controls [23]. The foundational model versions, underlying platform, and all settings were fixed throughout the study period.

#### Conversational Design and Rules

**Figure 1:**
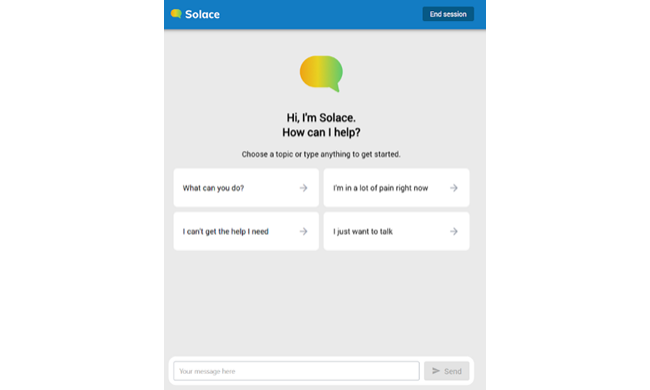
The user options for starting a Solace conversation, including the use of the free-text field or starter prompts.

Users began their experience with Solace by typing freely or selecting from starter prompts (Figure 1). User inputs were accepted without restrictions on length or content. Solace followed a set of core design principles developed through iterative collaboration with subject matter experts (SMEs) to ensure therapeutic integrity and user safety. These included the following:

1. Open-ended engagement, encouraging users to freely express their thoughts and emotions
2. Plain language to ensure accessibility and reduce cognitive burden
3. Emotional sensitivity, with empathic responses embedded throughout the conversation
4. Therapeutic guidance toward evidence-based strategies and self-understanding
5. Context continuity, using memory variables to support coherent, personalized dialogue
6. Multilevel safety guardrails, with screening for sensitive content (eg, mention of self-harm, harm to others, and opioid use) and fallback responses designed in collaboration with clinical experts.

**Figure 2:**
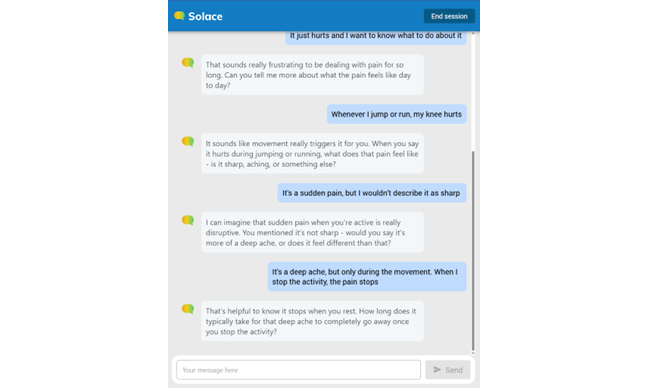
Examples of Solace outputs during a user conversation.

Solace’s outputs drew from knowledge bases composed from psychological frameworks, including but not limited to ACT, CBT, and dialectical behavior therapy and were presented to the user as (typically) 1-4 sentences (Figure 2).

#### Testing and Optimization

Initial development and testing were led by a clinical pain psychologist (PMS) and included a cohort of 8 SMEs, including other clinical psychologists, pain physicians, and pain researchers. SMEs conducted simulated therapy sessions with Solace and provided both real-time “think-aloud” feedback and postsession feedback to the development team, focusing on the conversational tone of Solace, therapeutic alignment, and emotional sensitivity. The development team worked with SMEs to translate their feedback into Solace’s conversational logic, which included refining pacing, language, and safety mechanisms.

Within Solace, user interactions are processed as string-based text inputs and routed through an orchestration layer that assigns each request to an appropriate specialized agent, which generates a response. Each specialized agent represents a specific element within the structure and therapeutic style of a clinical psychology session (eg, 1 agent for managing transitions between stages within a session), and has been fine-tuned and reviewed during development by an SME.

All generated responses are subsequently evaluated by a guardrail validation component that performs safety and compliance checks. If the validator fails, the response is discarded, and a specialized guardrail agent generates an alternative response (see “Safety and Errors” below); this agent and its outputs are likewise reviewed and refined by the SME. If validation succeeds, the approved response is returned to the user.

Stress-testing scenarios were used to explore how the system responded to ambiguous or sensitive inputs. System-level performance metrics, including response latency, session duration, and output length, were recorded during internal testing. This series of tests was part of an iterative process, informing and optimizing prompt development and the design of response templates.

#### Safety and Errors

System-level errors were defined as failure to generate a response, excessive latency, or loss of conversational flow. Errors were identified using a series of validator checkpoints embedded within the system architecture. Checkpoints initiated fallback mechanisms to trigger predefined safe response templates. System anomalies and platform reliability were further monitored using session-level logs and performance data. Detection of risks was supported by multilevel safety guardrails built into Solace’s conversation logic and prompt structures and relied on a combination of rule-based screening and automated classification techniques. The safety guardrails were designed to identify and mitigate risks in the following safety-sensitive domains: (1) disclosure of content indicating potential harm to self, suicidal ideation, or harm to others; (2) active psychological or medical emergencies; and (3) recommendations related to prescription medication use.

When high-risk content was detected, Solace generated predefined fallback responses designed to redirect the user to seek professional help, reinforce system boundaries, and discourage further engagement with unsafe topics.

### Participant Eligibility and Enrollment

Participants were recruited online through Prolific [24], an online research participation platform, and assigned an anonymous Prolific identification number. The target sample size for this study was 145 participants based on sufficiently approximating the population means across multiple continuous variables that are expected to have normal distributions. We aimed to recruit 195 participants to accommodate an anticipated 25% rate of attrition [25].

Those who consented were redirected to Gorilla [26], a cloud-based research platform hosting the questionnaires, to screen for eligibility and to access the survey. Participants were considered eligible based on the following criteria: (1) 18 years of age or older, (2) English speaking, and (3) living with chronic pain (ie, pain that has persisted for longer than 3 months). Chronic pain status and participant demographics were provided via the Prolific platform. Those eligible were asked to enter demographic information (age and sex) and to complete the baseline self-report measures.

### Chatbot Engagement

**Figure 3:**
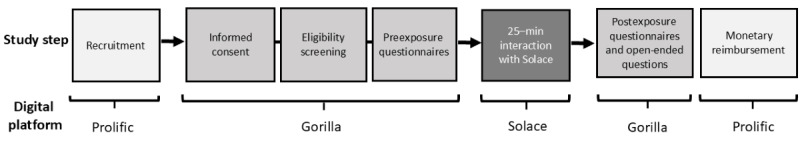
Participant flow through the study steps and associated digital platforms.

Participants were informed that Solace is a nonclinical investigational tool under development and is not intended to provide medical advice or replace professional care. Training or an opportunity for familiarization with Solace was not provided to the participants prior to the study. Participants were prompted to engage with Solace, which was described as a “conversational agent, developed by ManagingLife. Solace provides pain management support and evidence-based strategies to help you understand and manage pain in your daily life.” Participants were instructed to engage with Solace for at least 30 minutes from the start of the conversation, and then manually end the session. If users ended the session earlier than 30 minutes, they were informed how much time was left. Participants were then congratulated for completing their time with Solace and were provided with a link to return to Gorilla to complete the same self-report measures as at baseline, as well as a set of postexposure self-report measures. After completing the questionnaires, they were thanked for their participation, debriefed about the study hypotheses, and signposted to additional support if needed. The full participant flow through the study is depicted in Figure 3.

### Measures

#### Participant Demographics

Participant demographics, including sex, ethnicity, employment status, and pain duration, were obtained from Prolific records.

#### System Usability

The System Usability Scale (SUS) is a 10-item questionnaire designed to measure the usability of a system, including digital products or digital systems [27]. Participants are asked to respond to items regarding usability (eg, “I thought the system was easy to use”) on a scale from 1=strongly disagree to 5=strongly agree. The SUS is scored by subtracting the sum of the negatively worded items (eg, “I thought there was too much inconsistency in this system”) from the sum of positively worded items (eg, “I think that I would use this system frequently”), adding 20, and multiplying by 2.5 to get a score out of 100. An SUS score of ≥85 has been identified as “excellent.”

#### Treatment Acceptability

Two self-report inventories were included to examine treatment acceptability. The Treatment Acceptability/Adherence Scale (TAAS) is a 10-item self-report questionnaire designed to assess treatment acceptability and anticipated adherence to psychological interventions for anxiety and related disorders [28]. The TAAS has shown strong convergent and discriminant validity as well as excellent internal consistency [28]. In this study, the TAAS also demonstrated excellent internal consistency (α=.85). The Treatment Evaluation Inventory-Short Form (TEI-SF) is a 9-item measure of the perceived acceptability of behavioral treatments, and captures both positive (eg, “I would find this treatment to be an acceptable way of dealing with my chronic pain”) and negative (eg, “I believe this treatment is likely to harm or injure me”) perceptions of treatment [29]. The TEI-SF had excellent internal consistency in this study (α=.85). Both measures were adapted to the context of chronic pain treatment, which has been done in previous studies.

#### Therapeutic Alliance

The Working Alliance Inventory-Client Version (WAI-C) is a 36-item questionnaire used to measure therapeutic alliance in face-to-face therapy [30]. The WAI-C captures the client’s perspective on 3 elements of therapeutic alliance, including the bond between therapist and client, agreement on the goals of therapy, and agreement on tasks to accomplish those goals. The WAI-C has excellent internal consistency (α=.87) and is strongly related to psychotherapy treatment satisfaction and outcomes. It has also been shown to predict treatment response to therapeutic interventions for pain. This instrument was adjusted for use within the context of an interaction with an AI-agent to align with the study’s exploratory aims.

#### Pain-Related Measures

Patient Reported Outcomes Measurement Information System-29, version 2.0 (PROMIS-29) provides a profile of physical and mental health and assesses 7 health domains, including physical function, pain interference, pain intensity, anxiety, depression, fatigue, sleep disturbance, and social role integration. The PROMIS-29 has demonstrated strong validity in chronic pain populations. Internal consistency of subscales ranged from α=.88 to α=.96, indicating excellent consistency. The questionnaire was administered using the static short forms of the domains [31].

The Tampa Scale of Kinesiophobia (TSK) was included as a measure of fear of movement (and reinjury) [32]. Participants rate items relevant to this fear (eg, “Pain always means I injured my body”) on a scale from 0=strongly disagree to 4=strongly agree. The TSK has been extensively validated for individuals with chronic pain, and internal consistency in this study was adequate (α=.70)

The Pain Resilience Scale (PRS) is a 14-item measure of an individual’s ability to maintain positive physical and emotional functioning despite pain. Participants are asked to rate agreement to items (eg, “I push through the pain”) on a Likert scale from 0=not at all to 5=all the time [33,34]. The PRS has 2 subscales, behavioral perseverance and cognitive-affective positivity. The PRS has demonstrated psychometric validity among individuals with chronic pain [33], and internal consistency in this sample was excellent (α=.95).

The Chronic Pain Acceptance Questionnaire (CPAQ) is a 20-item measure with two subscales: (1) the degree to which one engages in life activities regardless of pain and (2) willingness to experience pain [35]. The CPAQ has been extensively validated for individuals with chronic pain [36,37], and the internal consistency in this study was adequate (α=.70).

#### System Metrics

User engagement and system performance were abstracted from transcripts of each participant session and associated data*.* User engagement characteristics included the number and length of prompts entered into Solace, the number and length of Solace’s responses, and total session duration. Response readability was calculated using the Flesch Reading Ease Score and the Flesch-Kincaid Grade Level [38,39]. Safety was assessed by manual review of all conversations that contained content relevant to the guardrails.

### Analysis

Analyses were primarily descriptive in nature. Participant responses on measures of usability, treatment acceptability, and therapeutic alliance were averaged (rather than summed) to give an indication of the overall level of agreement with measure items. Median (IQR) ratings for individual items were also reported. Averages with SD or counts, where applicable, were reported for system-level metrics. Only participants who completed at least 25 minutes of interaction with Solace were included for analyses of usability, treatment acceptability, and therapeutic alliance, whereas intent-to-treat analyses (eg, including all participants) were conducted using linear mixed effects models to analyze changes in clinically relevant self-report outcomes. Of note, due to a technical issue, 1 item was missed from the WAI-C (item 31) and 1 item from the CPAQ (item 8).

### Ethical Considerations

The University of Portsmouth Ethics Board approved this study (REB SHFEC 2025-073), and the study has been registered with the Open Science Framework. Written informed consent was obtained from all participants prior to taking part in the study. Participants were presented with information about the study, made aware of their right to withdraw from the study at any time, and provided consent. Participants were compensated £18 (approximately US $24) for completing the study.

All identifying information was removed from the data before storage, and the data were further anonymized by assigning ID codes. Data pertaining to participants’ responses to the structured and open-ended questionnaires and to their interactions with Solace were anonymized. Anonymized data were temporarily stored on the servers of Gorilla and ManagingLife, respectively. Data were extracted and transferred to the University of Portsmouth’s secure server. The data will be retained for a minimum of 10 years in accordance with the University of Portsmouth Retention Schedule for Research Data.

## Results

### Participant Characteristics and Flow

**Figure 4:**
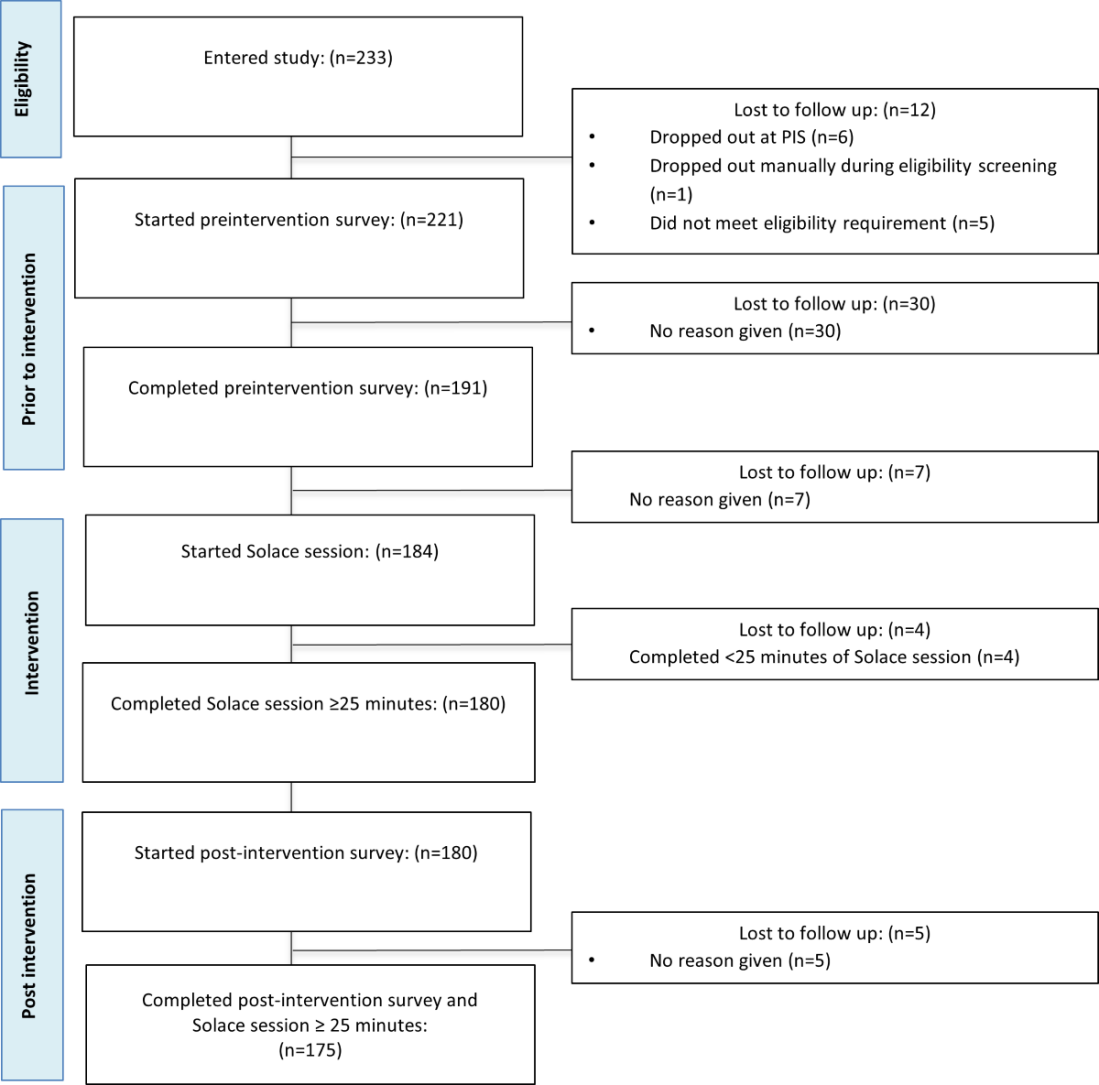
Flow diagram of participants who completed a ≥25-minute Solace session and self-report constructs. PIS: preintervention survey.

The flow of participants through the study is depicted in Figure 4. A total of 221 participants consented and completed the preinteraction survey. Of these, 184 began a session with Solace, and 175 engaged with Solace for at least 25 minutes. Participant demographics are listed in Table 1. All participants passed attention checks.

**Table 1:** Participant characteristics (n=175).

Characteristic			Value
Age (years), mean (SD)			42.54 (12.62)
**Sex, n (%)**			
	Male	81 (46.3)	
	Female	93 (53.1)	
	Prefer not to say	1 (0.6)	
**Ethnicity, n (%)**			
	Asian	12 (6.9)	
	Black	13 (7.4)	
	Mixed	9 (5.1)	
	White	139 (80.8)	
	Other	2 (1.1)	
**Employment status, n (%)**			
	Full time	93 (53.1)	
	Part time	28 (16.0)	
	Unemployed and job seeking	9 (5.1)	
	Not in paid work (eg, homemaker, retired, or disabled)	23 (13.1)	
	Other	8 (4.6)	
	No data	14 (9.7)	
**Pain duration, n (%)**			
	3-6 months	9 (5.1)	
	6-12 months	10 (5.7)	
	1-2 years	16 (9.1)	
	2-5 years	52 (29.7)	
	5-10 years	32 (18.3)	
	10-20 years	43 (24.7)	
	>20 years	12 (6.9)	
	No data	1 (0.6)	

### Usage Characteristics and Safety Performance

**Figure 5:**
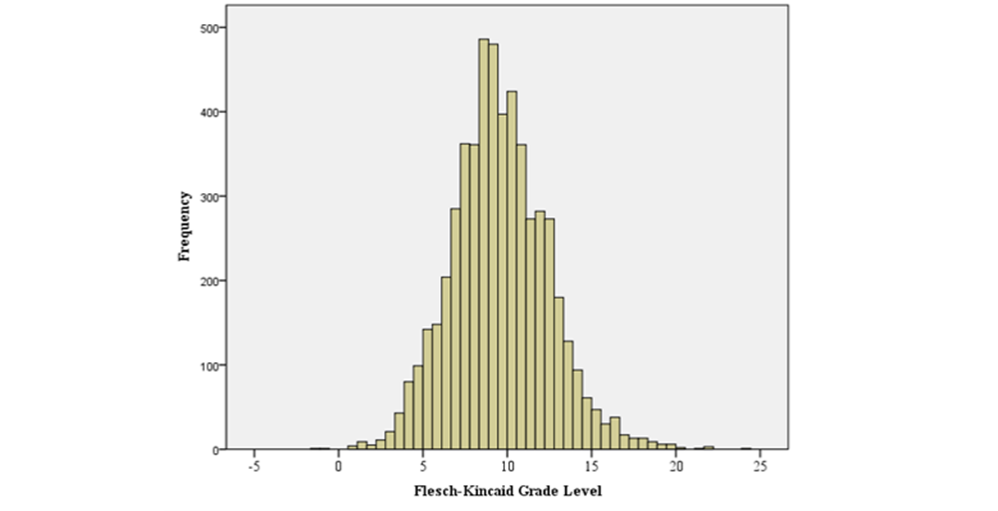
Distribution of Flesch-Kincaid Grade Levels across all responses generated by Solace.

**Figure 6:**
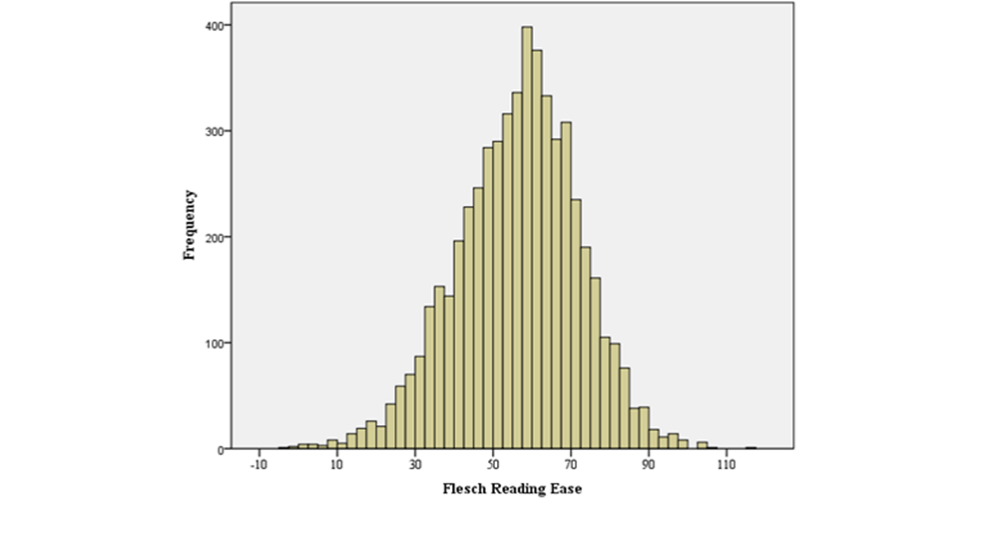
Distribution of Flesch Reading Ease scores across all responses generated by Solace.

The interaction sessions with Solace lasted an average of 38.74 (SD 11.7) minutes. On average, participants provided 29.2 (SD 10.1) input prompts to the system with an average length of 19.8 (SD 12.4) words, and Solace gave responses averaging 49.73 (SD 9.8) words on each occasion. On average, the readability of Solace’s responses was at the early high school level (Flesch-Kincaid Grade Level mean 9.56, SD 2.8; Flesch Reading Ease mean 56.44, SD 15.6). The distribution of Flesch-Kincaid Grade Levels and Flesch Reading Ease scores is depicted in Figures 5 and 6, respectively.

Notably, 2 transcripts featured discussions of death, and 89 transcripts featured discussions of medication. Upon review by a clinical health psychologist (PMS), neither instance where death was discussed constituted suicidal ideation, self-harm, or harm to others, and Solace’s responses were appropriate. Likewise, none of the conversations regarding medication included elicitation of recommendations, and, as such, guardrails were not activated.

### System Usability

The SUS was completed as a measure of how easy participants found Solace to use. Participants rated the usability of Solace as excellent (mean 85.04, SD 13.6). On a scale from 1=strongly disagree to 5=strongly agree*,* participants rated all items as “strongly agree” except “I would like to use this system frequently,” and “I found the various functions in this system were well-integrated,” which were both rated as “agree.” Item-level results are presented in Table 2.

**Table 2:** System Usability Scale item-level results (n=175). Scale ranges from 1=strongly disagree to 5=strongly agree.

Items	Median (IQR)
1. I think that I would like to use this system frequently	4.00 (2.00)
2. I found the system unnecessarily complex	1.00 (1.00)
3. I thought the system was easy to use	5.00 (1.00)
4. I think that I would need the support of a technical person to be able to use this system	1.00 (0.00)
5. I found the various functions in this system were well integrated	4.00 (2.00)
6. I thought there was too much inconsistency in this system	1.00 (1.00)
7. I would imagine that most people would learn to use this system very quickly	5.00 (1.00)
8. I found the system very cumbersome to use	1.00 (1.00)
9. I felt very confident using the system	5.00 (1.00)
10. I needed to learn a lot of things before I could get going with this system	1.00 (0.00)

### Treatment Acceptability

Participants completed 2 measures of treatment acceptability, the TEI-SF and the TAAS. Average scores for the TEI-SF and TAAS, as well as individual item characteristics (median, IQR), are presented in Tables 3 and 4, respectively. On a scale from 1=strongly disagree to 5=strongly agree, participants reported an average rating of 3.77 (SD 0.7) on the TEI-SF. On the TAAS, participants, on average, rated treatment acceptability as 5.47 (SD 0.92) on a scale from 1=disagree strongly to 7=agree strongly.

**Table 3:** Treatment Evaluation Inventory-Short Form item-level results (n=175). Scale ranges from 1=strongly disagree to 5=strongly agree.

Items	Median (IQR)
1. I would find this treatment to be an acceptable way of dealing with my chronic pain	4.00 (1.00)
2. I would be willing to use this procedure if prescribed or recommended by a physician	4.00 (1.00)
3. I like the procedures that may be used in this treatment	4.00 (1.00)
4. I believe this treatment is likely to be effective	4.00 (2.00)
5. I believe that I will experience discomfort during the treatment	3.00 (1.00)
6. I believe this treatment is likely to result in permanent improvement	3.00 (1.00)
7. I believe this treatment is likely to harm or injure me	1.00 (1.00)
8. I believe it would be acceptable to use this treatment with individuals who cannot choose treatments for themselves	4.00 (1.00)
9. Overall, I have a positive reaction to this treatment	4.00 (1.00)

**Table 4:** Treatment Adherence/Acceptability Scale item-level results (n=175). Scale ranges from 1=disagree strongly to 7=agree strongly.

Items	Median (IQR)
1. If I began this treatment, I would be able to complete it	6.00 (2.00)
2. If I participated in this treatment, I would adhere to its requirements	6.00 (2.00)
3. I would find this treatment exhausting	3.00 (3.00)
4. It would be distressing for me to participate in this treatment	2.00 (2.00)
5. Overall, I would find this treatment intrusive	2.00 (2.00)
6. This treatment would provide effective ways to help me cope with my chronic pain	5.00 (1.00)
7. I would prefer to try another type of psychological treatment instead of this one	3.00 (3.00)
8. I would prefer to receive medication for my chronic pain instead of this treatment	3.00 (2.00)
9. I would recommend this treatment to a friend with chronic pain	5.00 (2.00)
10. If I began this treatment, I would likely drop out	2.00 (3.00)

### Therapeutic Alliance

The WAI-C was completed to gauge the therapeutic alliance between participants and Solace. Overall, participants reported a strong working alliance with Solace (mean 188.03, SD 36.1), with a shared sense of goals rated as the strongest component of the therapeutic alliance (mean 67.86, SD 13.3), followed by agreement on tasks (mean 61.6, SD 12.7), and the therapeutic bond (mean 58.6, SD 12.6). WAI-C individual item scores are presented in Table 5.

**Table 5:** Working Alliance Inventory-Client Version item-level results (n=175). Scale ranges from 1=never to 7=always.

Items	Median (IQR)
1. I felt uncomfortable with Solace	1.00 (1.00)
2. Solace and I agreed about the things I will need to do in therapy to help improve my situation	6.00 (2.00)
3. I was worried about the outcome of the session	1.00 (1.00)
4. What I was doing in therapy gave me new ways of looking at my problem	5.00 (2.00)
5. Solace and I understood each other	6.00 (2.00)
6. Solace perceived accurately what my goals were	6.00 (2.00)
7. I find what I was doing in therapy confusing	1.00 (1.00)
8. I believe that Solace liked me	5.00 (2.00)
9. I wish Solace and I could have clarified the purpose of our session	2.00 (4.00)
10. I disagreed with Solace about what I ought to get out of therapy	1.00 (1.00)
11. I believe the time Solace and I were spending together was not spent efficiently	1.00 (2.00)
12. Solace did not understand what I was trying to accomplish in therapy	1.00 (1.00)
13. I was clear on what my responsibilities were in therapy	6.00 (2.00)
14. The goals of the session were important for me	6.00 (2.00)
15. I find what Solace and I were doing in therapy were unrelated to my concerns	1.00 (1.00)
16. I feel that the things I did in therapy helped me to accomplish the changes I wanted	5.00 (2.00)
17. I believe Solace was genuinely concerned for my welfare	5.00 (3.00)
18. I was clear as to what Solace wanted me to do in that session	6.00 (2.00)
19. Solace and I respected each other	7.00 (2.00)
20. I feel that Solace was not totally honest about its feelings toward me	1.00 (0.00)
21. I was confident in Solace’s ability to help me	5.00 (3.00)
22. Solace and I were working toward mutually agreed upon goals	6.00 (2.00)
23. I feel that Solace appreciated me	5.00 (3.00)
24. We agreed on what was important for me to work on	6.00 (2.00)
25. As a result of therapy, I became clearer as to how I might be able to change	6.00 (3.00)
26. Solace and I trusted each other	6.00 (3.00)
27. Solace and I had different ideas about what my problems were	1.00 (1.00)
28. My relationship with Solace was very important to me	4.00 (4.00)
29. I had the feeling that if I said or did the wrong thing Solace would stop working with me	1.00 (0.00)
30. Solace and I collaborated on setting goals for my therapy	6.00 (2.00)
31. I was frustrated by the things I was doing in therapy	—^a^
32. We had a good understanding of the kind of changes that would be good for me	6.00 (2.00)
33. The things that Solace was asking me to do did not make sense to me	1.00 (1.00)
34. I did not know what to expect as the result of my therapy	2.00 (3.00)
35. I believe the way were working with my problem was correct	6.00 (2.00)
36. I feel Solace cared about me even when I did things it did not approve of	5.00 (3.00)

^a^Not available.

### Clinical Outcomes

Clinically relevant outcomes were completed before and after the conversation with Solace and were analyzed using linear mixed effects models. Descriptive statistics and test results are presented in Table 6. Following Bonferroni correction to account for multiple comparisons, participants reported significant improvement in kinesiophobia, pain resilience, and pain willingness. On the PROMIS-29, participants reported improvement in anxiety and pain interference.

**Table 6:** Changes in clinically relevant outcomes.

Outcomes		Baseline, mean (SD)	Posttest, mean (SD)	*F* test (*df*)	*P* value
**PRS^a^**		28.07 (10.6)	30.57 (10.6)	41.62 (1, 193.6)	<.001
	PRS-Behavioral Perseverance	11.64 (4.2)	12.1 (3.9)	6.79 (1, 196.8)	.01
	PRS-Cognitive-Affective Positivity	16.43 (7.7)	18.48 (7.6)	51.95 (1, 194.1)	<.001
Kinesiophobia		43.4 (7.1)	41.79 (8.0)	24.73 (1, 191.5)	<.001
**CPAQ^b^**		61.66 (12.6)	62.5 (12.2)	1.71 (1, 201.1)	.19
	CPAQ-Activity Engagement	31.71 (8.0)	33.02 (8.1)	16.46 (1,194.05)	<.001
	CPAQ-Pain Willingness	29.95 (8.5)	29.48 (9.1)	0.37 (1, 194.05)	.54
**PROMIS-29^c^**					
	Physical Function	15.15 (3.5)	15.0 (3.6)	2.38 (1, 191.7)	.13
	Anxiety	10.46 (3.9)	10.0 (4.1)	17.04 (1, 191.2)	<.001
	Depressive Symptoms	9.58 (4.6)	9.42 (4.6)	2.82 (1, 192.2)	.09
	Sleep	12.87 (4.0)	12.86 (3.9)	0.01 (1, 193.6)	.93
	Social Function	13.0 (3.9)	13.08 (3.8)	0.37 (1, 195.1)	.55
	Pain Interference	11.60 (4.0)	11.15 (3.9)	10.89 (1, 195.1)	.001
	Fatigue	12.95 (4.1)	12.7 (4.4)	3.78 (1, 191.2)	.05

^a^PRS: Pain Resilience Scale.

^b^CPAQ: Chronic Pain Acceptance Questionnaire.

^c^PROMIS-29: Patient Reported Outcome Measurement Information System-29.

## Discussion

This study conducted a feasibility evaluation of Solace, an expert-trained generative AI conversational agent designed to use pain psychology–informed self-management strategies for chronic pain, following the DECIDE-AI framework [40]. Participants reporting chronic pain completed self-report measures before and after at least 25 minutes of conversation with Solace. Broadly, participants rated Solace to have excellent usability, good treatment acceptability, and reported a strong therapeutic alliance. Moreover, participants reported improvement in several clinically relevant domains from before to after the interaction with Solace.

The reported system usability (mean 85.04, SD 13.6) was rated well above the benchmark established for digital health applications (SUS score >65), indicating excellent usability [41,42]. Notably, SUS scores were also higher than those reported for other generative AI tools, such as ChatGPT, when rated by either general users (mean 67.44) or health care professionals (mean 64.52, SD 13.91) [43,44]. Although the SUS has been frequently used to evaluate AI applications [45], the SUS may not capture the nuance of using a generative AI conversational agent such as Solace [41]. Future studies should include more specific measures of usability designed to analyze generative AI conversational agents, such as the Bot Usability Scale [46].

Solace was deemed an acceptable health application for chronic pain and was well-received by participants. Participant responses on the TEI-SF and the TAAS were largely on par with other DHIs for chronic pain [47,48]. Participants responded with agreement to almost all items. This includes statements such as “I believe this treatment is likely to be effective,” “I would find this treatment to be an acceptable way of dealing with my chronic pain,” and “this treatment would provide effective ways to help me cope with my chronic pain.” Participants also endorsed items related to the belief that they could adhere to treatment with Solace, such as “If I began this treatment, I would be able to complete it,” and beliefs that Solace is a safe intervention (ie, strongly disagreeing with “I believe this treatment is likely to harm or injure me”). It is worth noting that participants rarely expressed the strongest agreement with the TEI-SF or TAAS items. This may be due to the noninterventional nature of the evaluation and the lack of set treatment expectations. It will be important to further evaluate acceptability in the context of a treatment trial or clinical practice.

Participants reported developing a strong therapeutic alliance with Solace over the course of the 25-minute conversation. In particular, participants reported a strong shared sense of treatment goals (ie, strongly disagreeing with the statement “I find what Solace and I were doing in therapy was unrelated to my concerns”). Several previous studies have examined the experience of therapeutic alliance with a conversational agent, and have found similarly strong working alliance [15,17]. It is perhaps understandable that participants reported the therapeutic bond as the weakest aspect of the alliance. Items designed to capture this on the WAI-C reflect a personal relationship that might not be relevant for interactions with a conversational agent, such as items asking if participants felt Solace liked or trusted them. New tools might be needed in this area to thoroughly assess therapeutic alliance with nonhuman conversational agents [49]. It is also notable that this study was not presented to participants as a treatment designed to help them with their pain, despite the conversational focus on treatment-related topics. It will be important to replicate these promising indications of therapeutic alliance with Solace in a treatment setting, and to examine if alliance improves with longer treatment duration, an important predictor of treatment success [50,51].

Safety of Solace and other generative AI conversational agents is a critical consideration in their development as DHIs. However, this could not be fully assessed as guardrails designed to manage suicidality, self-harm, medical emergencies, or medication recommendations were developed, but user prompts did not include safety concerns, and therefore, guardrails were not activated in this study. It will be necessary to analyze a much larger volume of interactions to fully test guardrail performance.

After the 25-minute conversation with Solace*,* participants demonstrated improvement in several meaningful clinical domains, including kinesiophobia, resilience, activity engagement, as well as anxiety and pain interference on the PROMIS-29 measure. Across these factors, there was 4%-9% improvement, with the greatest improvement seen in pain resilience. There are several difficulties in interpreting these changes. Notably, although participants were told Solace was a treatment in development and self-report constructs inherently included reference to treatment, they were also told that this was not a treatment study. There was also a mismatch between the wording on several of the measures and the time frame of the study. In particular, instructions for the subscales of the PROMIS-29 ask respondents to reference how they felt “in the last 7 days,” whereas the study might have lasted as little as 25 minutes. Despite these challenges in interpretation, improvements were all in the beneficial direction, held up to statistical correction, and likely reflect the participants' feelings at the time of responding. Although the change was modest in magnitude, this is a promising indication for further treatment-focused research on Solace*.*

Future research on Solace should take several directions. As described above, a program of clinical research should be undertaken to evaluate the efficacy and effectiveness of Solace, either as a stand-alone program or integrated into the Manage My Pain application, a digital pain self-management application developed by ManagingLife. This will include a fuller test of the effectiveness of the safety guardrails in identifying discussions involving recommendations for medication use, suicidal ideation, and harm to self or others. Further evaluation of the nature of users’ interactions with Solace will also be critical. These should focus on both the therapeutic relationship as well as evaluation of safety features of the application. Moreover, algorithmic fairness was not assessed in this study. Subsequent studies reporting on Solace will aim to compare model performance (sensitivity and specificity) across demographic subgroups, including sex, age, and ethnicity, to ensure equitable accuracy and lack of bias. Patient engagement is also needed to more fully address the lived experience of individuals with chronic pain.

There is room to further improve Solace’s functionality. In particular, Solace’s responses required a reading comprehension level considered roughly equivalent to early-middle high school. Future iterations of Solace should focus on the accessibility of language to improve utility by the general population. More detailed analysis of conversations with Solace is also warranted to ensure that conversational content is adherent to prompting.

There are several notable limitations of this study. First, the study was conducted via an online crowdsourcing platform. Although the sample recruited in this study is similar in several respects (ie, age, sex, and duration of pain) to individuals with chronic pain recruited from community samples [52], there were several notable distinctions, and also limited demographic data collection. In particular, participants in this study were primarily White and working full-time. As such, it is possible that community or treatment-seeking individuals with chronic pain would have a different experience of Solace. Although extensive research on chronic pain has been conducted via the Prolific platform [53,54], future research needs to be conducted in clinical samples with verified diagnoses. Second, the study was not designed as a test of Solace as a treatment. As such, participants did not necessarily have a treatment-related interaction with Solace, which could impact perceptions of acceptability and therapeutic alliance. Third, the sensitivity and specificity of the guardrail system were not fully tested, given the few instances that required guardrail activation. Finally, the short time frame of the study and the lack of a control condition limit the interpretation of the clinical impact of Solace. It is possible that alternative explanations, such as regression to the mean or social desirability, could account for the change associated with Solace use. Subsequent studies should examine prolonged or repeated interactions and feature comparisons to a control condition to enhance internal validity.

In conclusion, participants found Solace to be a usable system and an acceptable treatment for chronic pain after a 25-minute conversation. Participants reported a strong therapeutic alliance and demonstrated improvement in several clinically relevant domains. Furthermore, examination of safety guardrails for Solace indicated appropriate performance. Solace is a promising therapeutic tool, and clinical trials are needed to fully examine its clinical efficacy as a DHI for chronic pain.

## Data Availability

The data are available from the University of Portsmouth Research Portal, which functions as a data repository.

## Appendix

Multimedia Appendix 1 Developmental and Exploratory Clinical Investigation of Decision-Support Systems Driven by Artificial Intelligence (DECIDE-AI) checklist.

## Abbreviations

ACT: acceptance and commitment therapy
AI: artificial intelligence
CPAQ: Chronic Pain Acceptance Questionnaire
DECIDE-AI: Developmental and Exploratory Clinical Investigation of Decision-Support Systems Driven by Artificial Intelligence
DHI: digital health intervention
LLM: large language model
PROMIS-29: Patient Reported Outcomes Measurement Information System-29, version 2.0
PRS: Pain Resilience Scale
SME: subject matter expert
SUS: System Usability Scale
TAAS: Treatment Acceptability/Adherence Scale
TEI-SF: Treatment Evaluation Inventory-Short Form
WAI-C: Working Alliance Inventory-Client Version
TSK: Tampa Scale of Kinesiophobia
